# Technology of Manufacturing of ZC Cylindrical Worm

**DOI:** 10.3390/ma15186412

**Published:** 2022-09-15

**Authors:** Piotr Boral, Rafał Gołębski

**Affiliations:** Department Technology and Automation, Czestochowa University of Technology, Al. Armii Krajowej 21, 42-200 Częstochowa, Poland

**Keywords:** cylindrical worm, ball-end mill, helical surface, multipassing method, surface functional parameters

## Abstract

Cylindrical worms are generally machined by the hobbing method using rotary tools, and they are formed in the finishing pass at the full profile height. In this case, the profile of the tool-action surface determines the profile of the machined surface, and for technological reasons, a rectilinear (less frequently circular) axial profile of the tool-action surface is generally assumed. In the currently known technology, machining takes place on special machine tools, and on tools that are specially prepared for a specific outline. The research objective of the article is to present the possibility of creating a helical surface with a circular concave profile on a CNC lathe with a universal tool: a ball-end mill cutter. In the case of the proposed processing method, the surface of the worm is shaped with a spherical-end mill cutter in many passes, and its shape depends on the setting of the tool. This machining method must be performed on CNC machines, and the tool is not geometrically related to the shape of the machined profile. The paper presents the mathematical apparatus for generating a concave helical surface. Based on the calculations, the worm was processed with a spherical-end mill on a CLX350 V4 DMG MORI turning machining center. The surface-quality analysis was carried out on a contact profilographometer, while the dimensional accuracy was verified on a coordinate-measuring machine, and the maximum tolerance field of the measurement was 13 μm. On the basis of the measurements made, the accuracy of the worm outline is consistent with the theoretical assumptions. Using the presented method of machining, we can shape helical surfaces with an assumed profile in the axial section on a CNC machine tool with the use of universal tools.

## 1. Introduction

Cylindrical worms are most often made as worms with concave surfaces, or that are cone-derivative, and very rarely are they made as torus-derivative or others. In the literature, the construction and technology of worms with curved surfaces and cone-derivative worms are sufficiently well developed and described, while the other types of helical surfaces are described only sporadically and continue to be the subject of research. The helical surfaces of worms are made by the shaping and envelope method. When considering the issues of geometry and the manufacturing of worm gears, one should start with the works of Litvin [1]. The author presents the basics of the theory of the design, geometry, and production of all types of gears and gear drives. The publication of Radzevich [2] also presents a mathematical description of the geometry of the tools for machining various types of gears. The author proposes an innovative and practical mathematical method for designing gear-cutting tools. By narrowing the subject to worm gears, thanks to Litvin and Fuentes [3], the mathematical model of ZA-type hourglass worm gear was developed. The model of the ZN-type hourglass worm gear was described by Chen [4], based on its generation mechanism and the theory of gearing. The surface of the ZN-type hourglass worm is cut by a straight-edged cutting blade. In his work, Boral [5] describes the possibility of shaping the surface of the helical of the variable pitch, with a given profile, by milling the multipassing method. Besides fixed-pitch worms, variable-pitch worms are also applicable, and especially for use in polymer-processing machines. An analysis of the clearance between the threads of the worms in the system of two variable-pitched conical worms was introduced by Boral [6]. These worms are produced with a tapered cutter using special numerically controlled machine tools. To determine the size and distribution of the clearance at the thread height and length of the worm system, the evaluation of the accuracy of the contour and clearance measurement is carried out by the numerical method. 

CNC machine tools are often used in machining gear teeth. The technology of the machining of gear with an arbitrary profile, with the use of CNC machine tools, was presented in the investigation by Golebski [7,8]. He showed that the machining of gears with a tool that is not geometrically related to the contour being manufactured can be an excellent alternative to complicated and expensive hobbing methods. As well, a similar investigation was described by Kawasaki [9], based on tooth-contact analysis and worm gear-transmission errors, in which he prepared and carried out the machining of the worm by the end mill on a CNC machine tool. The tooth profiles of the worm wheel were modeled in a CAD system, with the model based on the analyzed results. The machining process was prepared in a CAM environment. The teeth of the worm wheel were also made by the end mill on a CNC machine tool.

In the envelope method for shaping the helical surface, different tool (milling cutter and grinding wheel) types, such as disc, shank, cup, and ring, are used. Whereas, in general, the tools shape the worm surface over the whole profile height, due to this, the tool profile for shaping a given helical surface is strictly defined and determined from the envelope condition, which has been described, among others, in the publications by Litvin [10] and Dudas [11]. Medvedev [12] presented an analysis of the shape of the tool for grinding different types of helical surfaces. Then, Skoczylas [13,14] showed the possibility of producing the concave thread of a worm formed by a tool with a straight profile. The geometry of the worm thread is made by a tapered-end mill. The developed method enables the easy machining of the concave profiles of worm thread by universal tools, and it also allows the machining of Archimedean worms. Mohan [15] has presented a rarely used machining method of the worm whirling process. A rotating tool ring encompassing the worm was used by this method. This machining is performed on a lathe. The authors showed the simulation of the whirling process, and an analysis of the effect of the tilting of the tool head. Zhang [16] also developed an interesting method of finishing the worm. The worm is machined using two honing wheels. This method improves the precision of machining and reduces the manufacturing cost.

In practice, a reverse task sometimes occurs. After identifying the worm (upon taking the measurements of its geometrical parameters and profile, if not given), high machining accuracy is needed. The form of the worm surface depends not only on the geometrical parameters of the tool and its positioning, but also on the tool type (such as the disc, finger, cup, or ring type), if it is able to finish shaping the worm convolution surfaces over the whole profile height. In practice, such an approach is difficult to reach because of the need for making appropriate calculations. For example, Popa [17] presents algorithms for profiling tools for milling and planning, in which the tool outline is defined by several points and could be expressed by a polynomial. It was assumed that the arbitrary profile worm would be cut with a ball-end mill, while the worm convolution flank would be shaped in multiple tool passes as the envelope of the partial helical surfaces. In their paper, Nieszporek et al. [18] discussed the theoretical shaping of a helical worm surface by several types of tools, with particular attention to machining with the use of a ball-end mill, but it was not performed in practice. This also applies to the publications presented by the authors in [19], as well as to those presented by [20], which included only general assumptions concerning the machining of the worm using a small-diameter milling cutter. This article presents the development of gear-wheel machining using the parametric programming of CNC machine tools. The work provides an excellent basis for the further development of the machining of worms by the multipassing method. 

Thus, the obtained resultant worm profile will have a multiangular profile, and it will not be a smooth curve. The same is true for, for example, the hobbing of spur gears or helical gears machined with a hob (the angularity depends on the number of blades on the tool perimeter), or with a Fellows cutter, for which the angularity depends on the number of enveloping tool positions. The literature provides relationships or diagrams for determining the nonsmoothness of machines profiles [19], although this fact is omitted in theoretical discussions.

A similar problem will occur in machining the helical surface by a ball-end mill after many tool passes. Thus, the number of passes and their distributions on the height of the machined surface profile should be selected so that the angularity of the profile and the deviations from the smooth surface of the preset worm helical surface profile fall within the permissible tolerance, or are in the order of magnitude of the surface-roughness height. Regarding this aspect, the work of Golebski and Boral [7] paid special attention during the machining of gears with the use a ball-end milling cutter. It can be assumed that the partial helical surfaces should be smooth, which results from the very high rotational speed of the ball-end mill compared with the speed of the tool’s relative helical motion. This is why finishing machining by milling is used more and more often. In a sense, a similar issue occurs in the machining of variable-pitch cone worms with a conical shank mill.

The helical surface quality prediction parameters are still an important research aspect in the area of analytical solutions for the construction of these surfaces. This was noted in the works by Pham et al. [21] and Xingqiao et al. [22]. The first of them presented a geometric model of the tool trajectory and its impact on the quality of the machined surface on CNC five-axis milling machining, while the second drew attention to the advantages of using CNC machines (in terms of quality) in the processing of complex surfaces, also on CNC machine tools.

After, it becomes necessary to grind the surface in the finishing process. Due to the technological difficulties of the process, grinding is often performed by manual processing with a fan or band-grinding wheels. In the machining method adopted in this work, the entire cutting process consists of many passes of the tool, which ensure high process productivity and high machining accuracy. With regard to the traditional methods of machining helical surfaces with circular axial contours, due to the technological difficulties and costs of making the tool, the machining of helical concave helical surfaces is rare. In the case of the adopted machining method, these limitations do not occur, and the form of the machined surface contour is irrelevant, which may result in the efficient machining of ZC-type worms. The multipassing machining technique is commonly used in machining gears and worms using a CAD/CAM environment. The mathematical model of machining a worm coil with any outline presented in this study allows for simplifying and shortening the time needed to prepare the technology for machining a coil. It eliminates the stage of the CAD modeling of the worm coil, as well as the stage of the preparation for machining in CAM, because the NC code is generated automatically based on the parameters of the worm, and it can be adapted to many machine-tool control systems. A very important aspect in the tested method is that the tool path does not depend on the accuracy of the generated model, as is the case with CAM systems. The surfaces of the solid geometry models are built of many triangles, and therefore, the generation of the machining paths is burdened with some inaccuracy, which does not occur in the analyzed case. 

In [23], the authors discuss the technological problems related to the principles of obtaining a high degree of precision in machining worm gear elements in order to eliminate backlash. 

In order to reduce the weight, vibrations, and noise, as well as resist corrosion, worm gears with plastic worm wheels are more and more often designed [24]. Such solutions are very often subject to low accuracy, and the gear is very often manufactured only by the injection method, without taking into account the finishing machining. The authors of [25] present research on the possibility of using polymeric materials with the addition of glass fiber to produce a worm wheel. The applied technology of reinforced polymer composites can be an excellent alternative to conventionally used materials.

Helical surfaces, and with particular emphasis on concave surfaces, are a very big technological challenge from the point of view of maintaining the high quality of the surface after machining. A number of factors, such as the constant changes in the inclination of the tool (in this case, the two-parameter changes) are of a significant nature in the subsequent research on the state of the surface structure. In the case of the mating surfaces (the worm and worm wheel), a 3D analysis of the functional parameters of the surface is necessary. The value of the functional parameters of the surface may approximate the expected operating time of the transmission, which was noted in the work by Krolczyk et al. [26]. 

The evaluation of the functional parameters of the surface may concern both the surface and volumetric parameters, as pointed out in the work by Szczotkarz et al. [27] presenting the three-dimensional topographic assessment of the surface. From the point of view of the nature of the work of the processed worm and the complexity of the profile being machined, the work includes research on the topographic analysis of the helical surface of the worm, which is necessary in this area, determining its volumetric functional parameters.

The paper presents the theoretical and practical aspects of the implementation and verification of a worm with a concave profile of the ZC type. In the second chapter, the mathematical and computational models of the created outline are presented, on the bases of which an algorithm for calculating the discrete values of the shape of the processed outline was developed, and consequently, the path (the trajectory of the tool) of the ball-end milling cutter. The calculations were implemented in practice in the machine-tool control program, and the contour of the worm was processed. In the next chapter, the result of the work was analyzed, the conformity of the outline was measured on a coordinate-measuring machine, and a detailed analysis of the topography of the machined surface—the concave helical coil—was manufactured. 

## 2. Materials and Methods

In comparison with traditional machining methods, in which the contour of the milling cutter depends on the contour of the helical surface to be machined, and the helical surface is machined along the entire height of the contour, the machining efficiency, in this case, is low, with high roughness of the machined surface. In the machining case presented in the work, the entire cutting process consists of many passes of the tool, it ensures high process productivity and high machining accuracy, and the helical surface is finished by milling. In the considerations, a finger tool with a circular outline in the axial cross section of the tool operating surface was used—a spherical-end mill. The accuracy of machining depends on the resolution of the tool paths at the height of the profile. The helix is formed by the resulting simultaneous rotation of the worm (around the Z axis), with the coupling of the tool movement along the axis of the worm. 

### 2.1. Selection and Description of Research Methodology

According to the ISO worm gears (worm-profile and gear-mesh geometry [28] standard), we can distinguish five types of worm geometries: type ZA—a rectangular worm in the axial section, also referred to as an Archimedean worm; type ZN—a rectangular worm in the normal section of the helical surface; type ZI—an involute worm with an involute axial profile in any normal section of the worm helical surface; type ZK (helicoid-conical)—formed by a conical surface at a selected rack angle with respect to the axial section of the screw; type ZC—with a concave (circular) axial profile, quite often called “cavex”. A technical report [28] also defines the methodology for machining the abovementioned outlines, with a particular emphasis on the technology used, including the setting of the tool itself. ZC worms are shaped with the disc tool type by grinding. Roughing machining is most often performed on a lathe with a tool with a rectilinear outline in the axial section. It should be noted that this is the general case of how the Archimedean-worm-type ZA is shaped. The technology of processing the worm with classical methods is laborious and subject to large uncertainty. A rotational grinding wheel with a circular acting surface wears out quickly during machining, which is especially exacerbated by the improper setting of the tool. Moreover, the method indicated in the technical report is not efficient. The research assumed the possibility of using a ball-end mill cutter tool that is not geometrically related to the outline of the machined worm. The proposed method has never been used in practice before. The authors developed their earlier research, contained in [18], describing the theoretical considerations of the creation of helical surfaces with different contours using the cup-type tool, disc-type tool, and finger tool. In the machining process, a numerically controlled machine tool was used (a lathe equipped with driven tools, and that was additionally simultaneously controlled in the C and Y axes), with the possibility of implementing the machining program in ISO code. The process of the parameterization of the machined surface takes place on the basis of the mathematical calculations of the tool movement with respect to the machined surface. The advantage of the method is great versatility. By introducing the proprietary software, the appropriate parameters of the ZC profile (the radius of the concave profile, the transition curves of bottom and head areas of the worm coil), we can very efficiently correct its shape. In the research assumptions, the experiment should confirm the high quality and accuracy of the machining, and it avoids the use of finishing processing methods.

### 2.2. The Mathematical Description of the Helical Worm Surface

**Figure 1:**
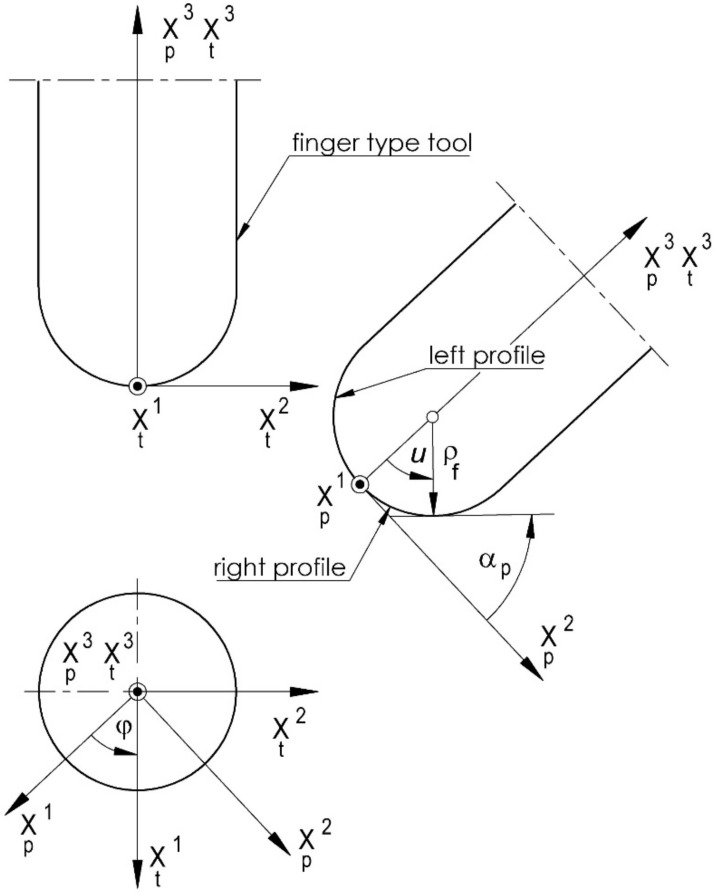
The form of the definition of the tool-action surface axial profile.

Description of the axial outline of the tool surface (Figure 1):

(1a) $$\langle -\begin{array}{cc} \frac{\pi }{2}, & \frac{\pi }{2} \end{array}\rangle ∋u\to \;\underset{p}{x}\left(u\right)={\left[\begin{array}{ccc} 0, & {\underset{p}{x}}^{2}\left(u\right), & {\underset{p}{x}}^{3}\left(u)\right) \end{array}\right]}^{T}\in E^{2},$$

(1b) $$\begin{array}{cc} m_{p}=\frac{\partial {\underset{p}{x}}^{3}}{\partial u}/\frac{\partial {\underset{p}{x}}^{2}}{\partial u}=tg\alpha , & \alpha \in \langle \begin{array}{cc} 0, & \pi \end{array}\rangle \end{array}$$

where $m_{p}$ is the slope of the tangent to the outline at a given point; *α* is the angle of the tangent; *u* is the parameter of the profile; *p* is the subscript identifying the coordinate system of the profile.

In a general case, this can be an arbitrary profile. For cone-derivative helical surfaces, this will be a straight line, while for torus-derivative helical surfaces, it will be a circular arc. If a rectilinear or circular profile is set in the specific section (axial, normal, or frontal), then the tool-action surface profile will be curvilinear, and it must be specially determined. In general, the worm profile may thus be an arbitrary curve (however, the normal to the worm axis may intersect the profile only once, which follows from the assumed machining technology).

However, in this discussion, it is assumed that the tool is a ball-end mill (Figure 1). The diameter of the mill is small, and smaller than the cut width of the worm core diameter. The tool must perform multiple passes to machine the whole profile. 

Upon a single pass of the tool (with helical movement) relative to the worm, a helical surface is obtained, which is a fragment of the worm helical surface being machined (hereafter, this surface will be called the partial helical surface, in contrast to the machined worm helical surface). By appropriately moving the tool relative to the worm being machined, another fragment of this surface will be shaped in the subsequent pass (if there are infinitely many such passes, then the worm helical surface and the partial helical surface contact one another along the helix, which is only at one point in an arbitrary section). After appropriately shifting the tool in relation to the worm profile in successive (multiple) passes, the machined worm helical surface will be obtained:

(2) $$\underset{t}{x}\;\left(\begin{array}{cc} u, & \varphi \end{array}\right)=\left[\begin{array}{cc} 3, & \varphi \end{array}\right]\;\underset{p}{x}\;\left(u\right),$$

where *φ* is the parameter (angle of rotation of the profile around the tool rotation axis) of the tool-action surface, and *u* is the parameter of the profile (parameter for the position of a point on the tool outline).

In Relationship (2), the following denotation is adopted for the matrix of elementary rotation (around the coordinate-system axes) around the axis with the number (*n*) by the angle *:

(3) $$\left[M\right]=\left[\begin{array}{cc} n, & * \end{array}\right]=\left[\begin{array}{ccc} m_{11} & m_{12} & m_{13}\\ m_{21} & m_{22} & m_{23}\\ m_{31} & m_{32} & m_{33} \end{array}\right],$$

Considering the rotation around the axis that coincides with one of the coordinate-system axes, the rotation-axis versor can be written as follows:

(4) $$e={\left[\begin{array}{ccc} e_{1}, & e_{2}, & e_{3} \end{array}\right]}^{T}{\left[\begin{array}{ccc} \delta_{1n}, & \delta_{2n}, & \delta_{3n} \end{array}\right]}^{T},\;n\in \langle 1,2,3\rangle$$

where $\delta_{in}$ is the Kronecker delta, while the subscript n identifies the axis of rotation. Thus, the denotation of the matrix of the elementary rotation around one of the coordinate-system axes, as defined by the number in Form (3), is unique. In the developed computation program, the rotation-matrix elements are calculated from the relationship:

(5a) $$m_{kk}=e_{k}^{2}\left(1-\cos*\right)+\cos*$$

(5b) $$m_{ij}=e_{i}e_{j}\left(1-\cos*\right)-\varepsilon_{ijk}e_{k}\sin*$$

where *m* is the elements of the matrix (3); *i*, *j* are the subscripts denoting, respectively, the matrix row and column numbers (*i*, *j*, *k* = 1, 2, 3); $\varepsilon_{ijk}$ is the Levi-Civita symbol.

In the prepared model, the hob offset from the helical-surface axis by the shift in the cut in the axial direction needs to be considered:

(6) $$\underset{b}{x}\;\left(\begin{array}{cc} u, & \varphi \end{array}\right)=\;\underset{t}{x}\;\left(\begin{array}{cc} u, & \varphi \end{array}\right)+\left[\begin{array}{ccc} r_{o}, & 0, & f_{o} \end{array}\right],\;n\in \langle 1,2,3\rangle ,$$

**Figure 2:**
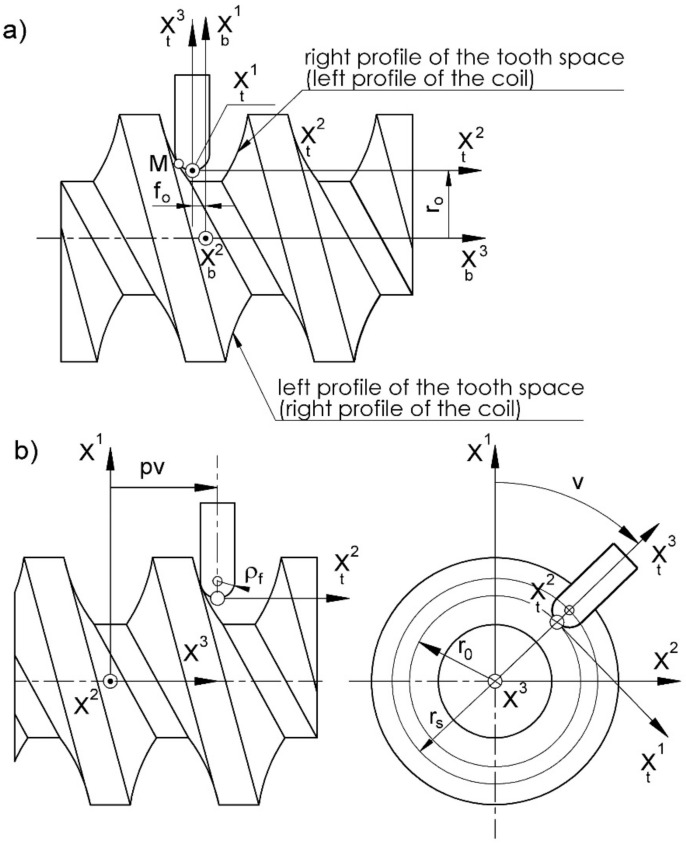
(**a**) Positioning and (**b**) relative helical motion of the mill and machined worm surface.

This is shown in Figure 2.

After considering the positioning and relative helical motion of the tool and worm (Figure 2), the following equation of the family of tool-action surfaces (moving surfaces) in the worm system is obtained:

(7) $$x\;\left(\begin{array}{ccc} u, & \varphi , & v \end{array}\right)=\left[\begin{array}{cc} 3, & -v \end{array}\right]\;\left\{\begin{array}{c} \left[M\left(\varphi \right)\right]\;\underset{p}{x}\;\left(u\right)+\\ +{\left[\begin{array}{ccc} r_{o}, & 0, & f_{o} \end{array}\right]}^{T} \end{array}\right\}+{\left[\begin{array}{ccc} 0, & 0, & \pm pv \end{array}\right]}^{T}$$

(8) $$\left[M\left(\varphi \right)\right]\;=[\begin{array}{cc} 3, & -\frac{\pi }{2} \end{array}]\;[\begin{array}{cc} 1, & -\frac{\pi }{2} \end{array}]\;\left[\begin{array}{cc} 3, & \varphi \end{array}\right]$$

where *v* is the parameter of the relative worm helical and tool motion; *p* is the parameter of the surface; $r_{o}$ and $f_{o}$ are the tool positioning parameters (Figure 2); ± is for the right-hand and left-hand worm, respectively.

This is a fragment of the worm helical surface, which is generated on a single tool pass. 

Surface-family Equation (7) is a function of three parameters, while the surface is a function of two parameters. Therefore, it is necessary to enclose the envelope condition, which can be generally written in the form of the triple product (scalar and vector products) of three vectors:

(9) $$f_{1}\left(\begin{array}{ccc} u, & \varphi , & v \end{array}\right)=x_{u}x_{\varphi }x_{v}=0\;$$

where the designation $x_{•}=\frac{\partial x}{\partial •}$ is taken.

The envelope condition, after transformations, can be written in the following form:

(10) $$f_{1}\left(\begin{array}{cc} u, & \varphi \end{array}\right)=\left\{\begin{array}{c} {\left[M\left(\varphi \right)\right]}^{T}\left(-\left[3\right]\;\left(\begin{array}{c} \left[M\left(\varphi \right)\right]\underset{p}{x}\left(u\right)+\\ +{\left[\begin{array}{ccc} r_{0}, & 0, & f_{0} \end{array}\right]}^{T} \end{array}\right)\right)+\\ +{\left[\begin{array}{ccc} 0, & 0, & \pm p \end{array}\right]}^{T} \end{array}\right\}\cdot c\;\left(u\right)=0$$

(11) $$c={\left[\begin{array}{ccc} 0, & 1, & m_{p} \end{array}\right]}^{T}\times \left(\left[3\right]\;\underset{p}{x}\;\left(u\right)\right)$$

The envelope condition does not include the v parameter of the surface family.

The worm axial profile can be determined from the condition (from this equation, the value of the v parameter is determined, while the value of the *φ* parameter is already determined from the envelope condition):

(12) $$x^{2}=0\Rightarrow v\;\left(\begin{array}{cc} u, & \varphi \end{array}\right),$$

**Figure 3:**
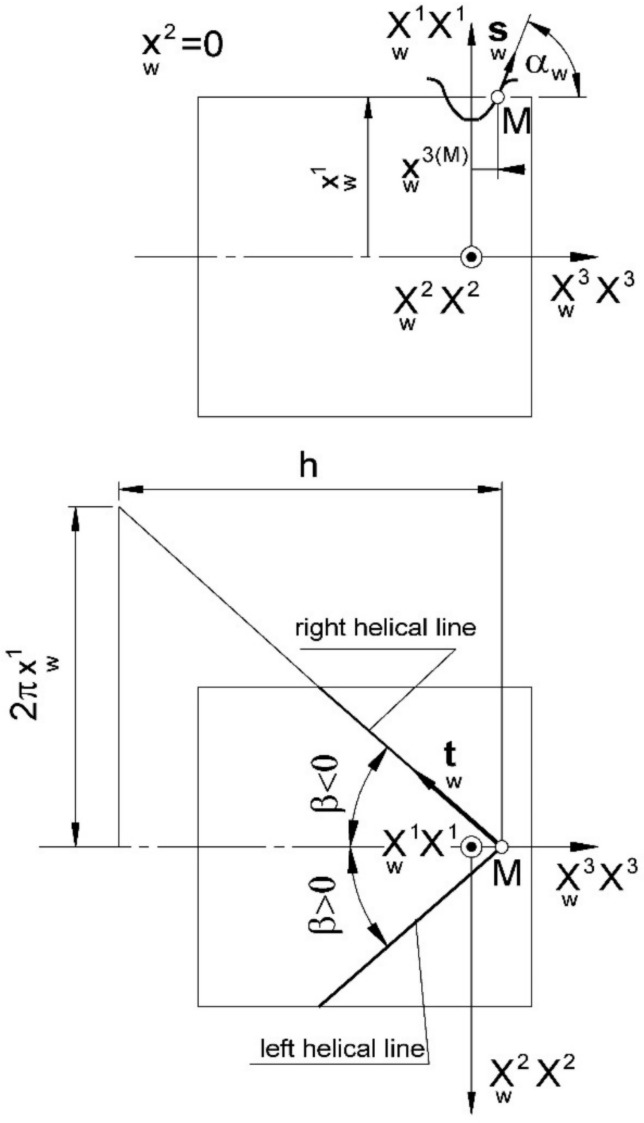
The form of setting the helical-surface profile.

Equations (7) and (10) describe the worm surface, but by having the axial profile of this surface, the surface can be determined by moving the profile along a helix:

(13) $$x=\left[\begin{array}{cc} 3, & -v \end{array}\right]\;\underset{w}{x}+{\left[\begin{array}{ccc} 0, & 0, & \pm pv \end{array}\right]}^{T},$$

where, in contrast to (7), $\underset{w}{x}$ denotes the worm axial profile (either preset or calculated, as above), and also in these equations, the v parameter of the helical surface has a slightly different interpretation (from in (7)), where it is the parameter of the relative tool and worm motion, while in (13), it is the parameter of the helical “motion” of the worm axial profile. Using (12), the worm axial profile is determined, but by taking into account the parallel shift of the cutting plane and determining the worm profile in these planes (Figure 3).

(14) $$x^{2}=s\Rightarrow v\;\left(\begin{array}{cc} u, & \varphi \end{array}\right),$$

where the parameter *s* denotes the position of the cutting plane (the distance of the cutting plane from the worm axial plane, as measured in the $X^{2}$ axis direction).

### 2.3. Determining the Mill Positioning Parameters

**Figure 4:**
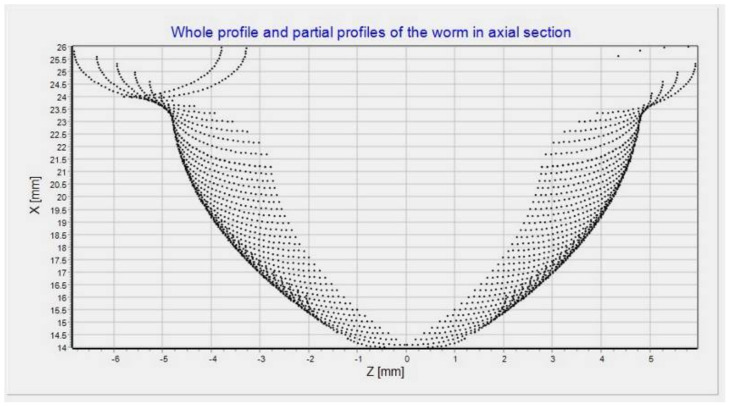
Authors’ program window: worm profile and profiles of partial helical surfaces in axial section: module: *m* = 5; outer diameter: $d_{1}=48$ (mm); *k* = 1 number of threads; number of passes = 50; worm axial profile radius: ρ=15 (mm); milling cutter radius: $\rho_{f}=2$ (mm).

Above, the determination of the worm helical surface for the present tool profile (being arbitrary in a general case; in the case under consideration, this was a circular profile) is described. The worm surface will be obtained as a set of those surface fragments after multiple tool passes (Figure 4). The drawing shows the formation of the axial contour of the worm, illustrated by the successive setting of the axial contour of the tool operating surface, marked with dotted lines. The axial outlines of the parts of the spherical surface of the tool for subsequent settings relative to the worm create the axial section of the worm. Thus, one can be sure to specify the values of the parameters $r_{o}$ and $f_{o}$ for the tool positions (mill or grinding wheel) for the set worm axial profile.

Similar to the above, the worm surface of the machined worm is described in the $\underset{w}{x}$ system in the axial cross section, as well as the tangential directional coefficients at these points (Figure 3):

(15a) $$\underset{w}{x}=\left[\begin{array}{ccc} {\underset{w}{x}}^{1}, & 0, & {\underset{w}{x}}^{3} \end{array}\right]{\;}^{T}$$

(15b) $$m_{w}=tg\alpha_{w}$$

where $\begin{array}{cc} {\underset{w}{x}}^{1}, & {\underset{w}{x}}^{3} \end{array}$ are the coordinates of the machined workpiece profile points in the axial plane of the worm; $\alpha_{w}$ is the angle of the tangent; $m_{w}$ is the slope of the tangent.

The lead angle at any point of the worm axial profile (depending on the point positioning angle (${\underset{w}{x}}^{1}$)) is (Figure 3):

(16) $$\beta =\pm arctg\frac{{\underset{w}{x}}^{1}}{p},$$

The versor of the tangent to the helix line in the profile system ($\underset{w}{X}$) at the profile point under consideration lies in the plane:

(17) $${\underset{w}{X}}^{1}={\underset{w}{x}}^{1},$$

and is (Figure 3):

(18) $$\underset{w}{t}=\cos\beta \;\left[\begin{array}{ccc} 0, & tg\beta , & -1 \end{array}\right]{\;}^{T},$$

The versor tangential to the worm axial profile (Figure 3) is:

(19) $$\underset{w}{s}=\cos\alpha_{w}\;{\left[\begin{array}{ccc} m_{w}, & 0, & 1 \end{array}\right]}^{T},$$

The versors $\underset{w}{t}$ and $\underset{w}{s}$, both determined at the same point, lie in the plane tangential to the helix at a given point. Therefore, in the considered points of the axial cross section of the worm, the normal versor of the helical surface can be determined as the vector product of the above versors:

(20) $$\underset{w}{n}=\pm \frac{cos\beta }{\sqrt{{sin}^{2}\beta +{\left(m_{w}\right)}^{2}}}{\left[\begin{array}{ccc} tg\beta , & -m_{w}, & -m_{w}\;tg\beta \end{array}\right]}^{T},$$

where (+) is the left side of the worm thread, and (−) is the right side of the worm thread.

It follows from the envelope condition that, at the contact points of the tool-action surface and machined surface, the normals to these surfaces coincide. From Relationship (18), the normals were determined for the successive worm axial profile points lying on the surface. In the case at hand, the tool is a ball-end mill with a spherical end. Thus, from the normal to the tool-action surface passes through the center of the mill’s spherical end. 

A segment of the spherical surface of the ball-end mill shapes the partial channel helical surface. Then, the helical surface is formed from many partial surfaces as a result of the many passes of the mill. The curve of the momentary contact of the tool-action surface with the envelope is an arc that coincides with the axial profile of the tool, and it lies in a plane perpendicular to the helix of the trajectory of the sphere-center movement in the worm system.

Equation (1a) is as follows:

(21) $$\underset{p}{x}\;\left(u\right)=\rho_{f}{\left[\begin{array}{ccc} 0, & \sin u, & 1-\cos u \end{array}\right]}^{T},$$

Based on Relationships (15) and (16), it is possible to determine:

(22) $$\varphi_{c}=k\frac{\pi }{2}\pm arctg\frac{r_{0}+\rho_{f}}{p},\;k=0\vee 1$$

where (+) is the right side of the worm thread; (−) is the left side of the worm thread; *k* is equal to 0 or 1 for the right-hand or left-hand helix, respectively.

For the given parameter (*φ*), the partial helical surface can be described by a helical movement of the tool characteristics in a worm system, with two-parameter Equation (7):

(23) $$x\;\left(\begin{array}{cc} u, & v \end{array}\right)=\left[\begin{array}{cc} 3 & -v \end{array},\right]\left\{\left[M\left(\varphi_{c}\right)\right]\;\underset{p}{x}\left(u\right)+{\left[\begin{array}{ccc} r_{0}, & 0 & f_{0} \end{array}\right]}^{T}\right\}+{\left[\begin{array}{ccc} 0, & 0, & \pm pv \end{array}\right]}^{T}$$

During machining, forming a normal-to-workpiece profile at the point of contact of the circular-profile tool will pass through the center of its circular profile:

(24) $${\underset{w}{x}}^{\left(T\right)}=\underset{w}{x}+\underset{w}{n}\rho_{f}$$

where ${\underset{w}{x}}^{\left(T\right)},\underset{w}{x}$ are the coordinates of the points of the spherical-tool-end center (the superscript identifies the tool) and the points of the axial profile of the worm helical surface, respectively, and $\rho_{f}$ is the tool profile radius.

The relative positions of the tool and worm at the time of shaping successive worm axial profile points will be different. The distances of the ball-end-mill spherical-end center points from the worm axis at the time of shaping the successive worm axial profile points can be defined from the relationship (Figure 2):

(25) $$r_{s}=\sqrt{{\left({\underset{w}{X}}^{1}{}^{\left(T\right)}\right)}^{2}+{\left({\underset{w}{X}}^{2}{}^{\left(T\right)}\right)}^{2}}\Rightarrow r_{o}=r_{s}-\rho_{f}$$

Relationship (25) does not have the third component described by Relationship (24). The angle founded between this radius and the axial plane is equal to:

(26) $$\psi =arctg\;\frac{{\underset{w}{X}}^{2}{}^{\left(T\right)}}{{\underset{w}{X}}^{1}{}^{\left(T\right)}}$$

where *ψ* is the angle formed by the normal to the machined surface passing through the center of the arch forming the profile of the tool with the axis of the worm.

The helical surface can be described with the helical movement of its arbitrary profile (it can be a characteristic), which is illustrated by the relative helical motion of the $\underset{w}{X}$-coordinate system that is rigidly related to the contemplated contour in relation to the stationary reference system (*X*). At the initial moment, both coordinate systems coincided. The relative helical motion of the tool and worm during machining can be described analogously to Equation (13). If the points of the spherical end of the tool are transferred to the plane of the axial contour of the worm, then the values of the parameters $r_{0}$ and $f_{0}$ for the tool settings for the worm machining that appear in Equations (7) and (10) can be obtained (additionally taking into account the radius of the cutter contour). Thus, the coordinates of the tip of the end mill, after being transferred by a helical movement to the axial plane of the worm (analogous to Figure 2 and Equation (13)) will be equal to:

(27) $$x=\left[\begin{array}{cc} 3, & \psi \end{array}\right]\;{\underset{w}{x}}^{\left(T\right)}+\left[\begin{array}{ccc} 0, & 0, & \pm p\psi \end{array}\right]{\;}^{T}+{\left[\begin{array}{ccc} -\rho_{f}, & 0, & 0 \end{array}\right]}^{T}$$

where ± is for the right and left sides of the worm helix, respectively.

**Figure 5:**
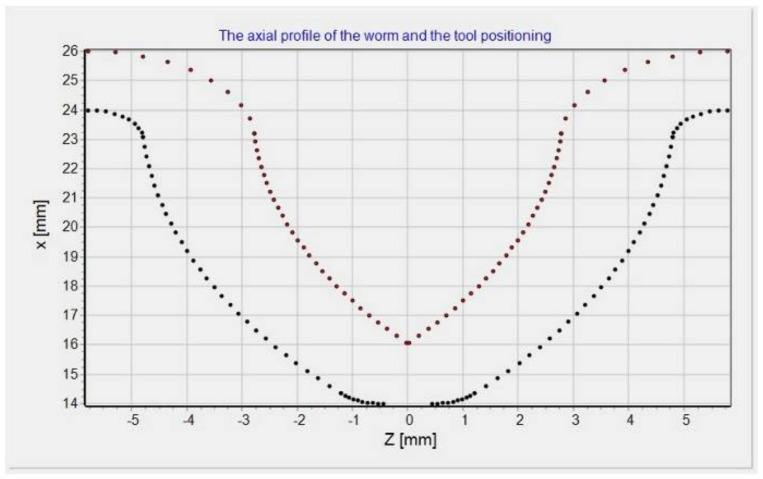
Authors’ program window: the points of the successive positions of the tool center, and the points of its contact with the axial contour of the worm with the following tool positioning parameters: module: *m* = 5; outer diameter: $d_{1}=48$ (mm); *k* = 1 number of threads; number of passes = 50; worm axial profile radius: ρ=15 (mm); milling cutter radius: $\rho_{f}=2$ (mm).

The points sought were determined in the equation shown in Figure 5:

(28) $$x={\left[\begin{array}{ccc} r_{o}, & 0, & f_{o} \end{array}\right]}^{T}={\left[\begin{array}{ccc} X, & 0, & Z \end{array}\right]}^{T}$$

Figure 5 shows two series of points: the first one above shows the successive positions of the tool (center of the spherical surface), while the second sequence of points below shows the points of the spherical surface of the tool with the surface of the worm coil in the axial section. The presented graphs in Figure 4 and Figure 5 were generated from a proprietary program for the automatic generation of a control program for a CNC machine tool for machining a helical surface with a curvilinear profile.

The radius tool profile should be selected to enable the convolution-transition-curve radius to be machined into the cut bottom, or:

(29) $$\rho_{f}\leq 0.35m_{o}$$

where $m_{o}$ is the axial module.

### 2.4. Machining Process

Based on the analysis of the discrete record of the coordinates, a computation program that controls for worm machining on a CNC lathe controlled in 4 axes (x, z, y, c) was developed. The used DMG MORI CLX350V4 lathe is an underused machine with a total work time of 150 h of the spindle. The machine tool provides a very high repeatability of machining, in accordance with the results of DMG MORI accuracy test results, based on the ISO standard [29], and the straightness of movement along the length of 300 mm in the ZX plane is 0.005 mm, and in the YZ plane, it is 0.003 mm. The parallelism of the movement of the Z axis and the tailstock spindle axis at a length of 60 mm from the spindle in the ZX plane is 0.001 mm, while in the YZ plane, it is 0.002 mm. The machine tool is mainly used for research (experimental and didactic processing). 

By extending the coordinates that define one plane of the tooth profile in the direction of the width of the worm in the helical direction, it is possible to generate a solid model of the machined worm to verify the correctness of the calculations. The tool path can be saved in a separate text file, in which the tool diameter was taken into account. In the calculation program, the generated outline includes separate resolutions of the transitions of the root-area tool and the useful area of the outline (the concave line and outline head, respectively). In the case of the ball-end mill, its positioning at the lead angle of the worm thread is not considered, although this needs to be taken into account when using other rotary tools (such as the disc, ring, and cup types).

**Figure 6:**
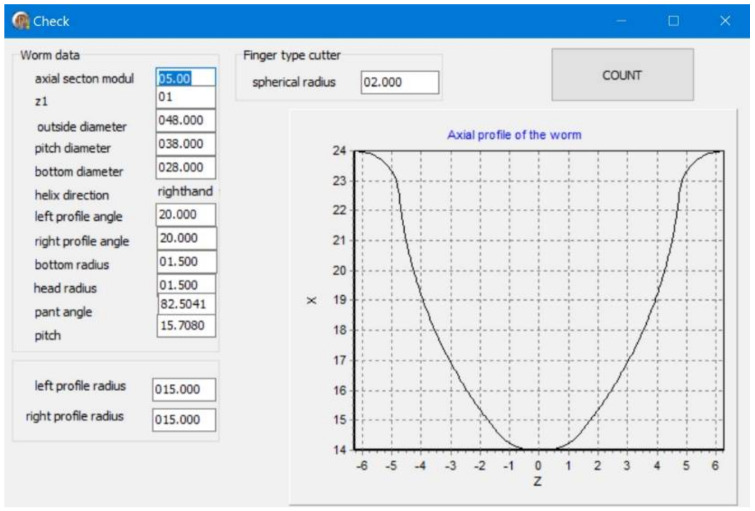
Authors’ program window: the parameters set and previously calculated, and a graphic showing the calculated axial profile of the worm for generating a control code for finishing the screw on a CNC machine tool.

In order to make the computation, the basic parameters of the worm (that is, the module, number of threads, length, outer diameter, pitch diameter, cut bottom diameter, axial-profile angle, thread direction, and addendum and thread root-rounding coefficients), and of the tool, should be given. On their bases, the worm cut axial profile is calculated (Figure 6).

**Figure 7:**
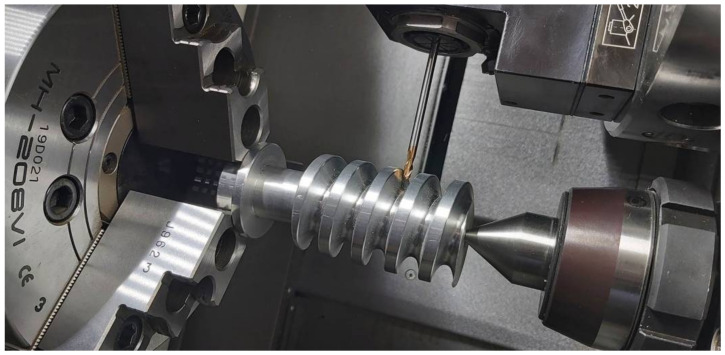
The machined worm mounted in a three-jaw chuck (CNC lathe: DMG MORI CLX350V4).

A numerically controlled lathe from DMG MORI, model CLX350V4, equipped with a Sinumerik 840D sl control system, was used for machining. The blank was made of a uniform AlSi1MgMn aluminum material, mounted in the three-jaw self-centering chuck of the lathe (Figure 7). The Mahr MarCator 1075R (Mahr GmbH, Göttingen, Germany) measuring sensor, with a measuring resolution of 5 μ and a reading resolution of 1 μ, was used to check the correct mounting (the parallelism of the axis of the blank part to the lathe spindle (*Z* axis)). By adjusting the clamping force of the jaws, it was possible to obtain a parallelism deviation in the *Z* axis on a length of 120 mm of 8 μ. For the machining, a ball-end mill cutter (VHM) with high accuracy was used. 

The machining was divided into two stages: roughing and finishing. The roughing machining was carried out using an 8 mm-diameter ball-end mill, with an assumed 30 passes per worm on the cut flank side. On account of the cut width, the tool made 18 passes per profile side. A 0.2–0.25 mm allowance for the finishing machining was left. 

The finishing machining was performed using a 4 mm-diameter ball-end mill with 50 passes per worm on the cut side. A rotational speed was adopted at 5000 rpm, and it was limited by the capabilities of the driven toolholder. 

**Figure 8:**
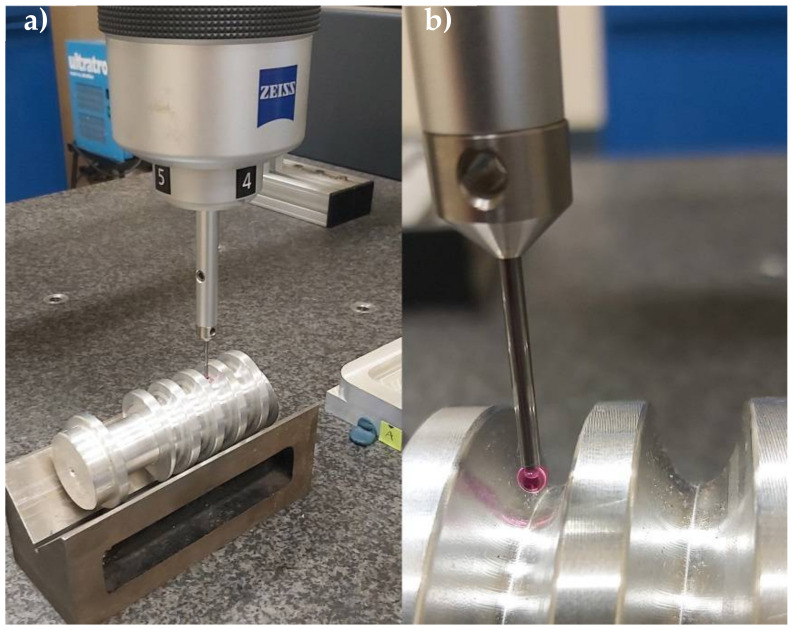
Measuring of concave profile on CMM Zeiss Eclipse: (**a**) worm fixed on the prism; (**b**) setting the measuring tip in the axial section of the worm.

As a rule, in-cut milling is used in finishing milling, but in the case of some materials, (e.g., composite materials), better roughness parameters are obtained in up-cut milling. In order to check the impact of the in-cut and out-cut machining strategies on the condition of the machined worm surface, the right-side-surface machining of the coil was performed by down-machining, and the left side was performed by up-machining. On the surface treated with up-milling in the central part, there was surface snagging, which can be seen in Figure 8. This was caused by the more difficult working conditions of the spherically ended tool during the machining of the concave surface in the place where the arc of contact became larger.

The aspect of the squareness of the surface after machining with an end mill is very important because it directly affects the size of the deviations from the theoretical outline. In theory, an infinite number of passes are required to achieve a smooth point-shaped surface. The number of passes of the tool and its contour determine the roughness and accuracy of the helical outline. Therefore, the number of passes at the height of the screw profile should be selected so that the theoretical prism is within the assumed allowable tolerance of the worm-profile errors. In the case of a concave worm profile, the number of passes of the tool with a spherical profile in relation to convex or rectilinear profiles is smaller. 

In the considered technology of worm processing, in simplified terms, for any concave contour, with regard to the section in question, the circular-concave curvilinear contour is replaced by an arc with a smaller radius mapped by the tool. The research and relationship in this area have already been presented by the authors as general research in previous articles [5,7,19]. Due to the productivity of machining, the worms are processed successively with tools of larger and smaller diameters.

## 3. Results

In the case of the ball-end mill, its positioning at the lead angle of the worm thread is not considered, although this needs to be taken into account when using other rotary tools (such as the disc, ring, and cup types). The machining of concave curves is technologically very demanding. The planned allowance for the depth of the cut in the finishing machining may change to a small extent, and this is caused by the squareness of the profile obtained after roughing. The greater challenge is to change the tool inclination in the workpiece. This is due to changes in the tangency of the tool profile when machining concave curves. The change in the cutting width by the spherical-end tool causes its vibration, and thus, the above normative deterioration of the surface-roughness profile.

Therefore, it is reasonable to verify the consistency of the cut worm axial profile with the geometric parameters set in the program. Measurements of the worm axial concave profile were taken on a Zeiss Eclipse coordinate-measuring machine (see Figure 8).

When checking the contours of the gears, with particular regard to the profiles of the worms, the accuracy is assessed using specialized ZEISS GearPro software. The idea of the measurement is to determine the normalized deviations (the detailed geometrical parameters of the outline in relation to the nominal outline calculated by the software). This was presented in Golebski’s works [6,7]. Control of the ZC worm profiles is more complex. A concave profile is not a standard profile, and hence, it is not possible to make standardized measurements. In the case under study, the analysis covered the compliance of the contour (its circularity in the axial cross section in the axis coordinate system of the worm helical surface). The examined worm was fixed on the prism (Figure 8a), and then, after determining the axial section of the worm (setting the measuring tip (Figure 8b)), the measurements were made for 60 points of the profile. The measurement was repeated 10 times for different cross sections of the contour.

**Figure 9:**
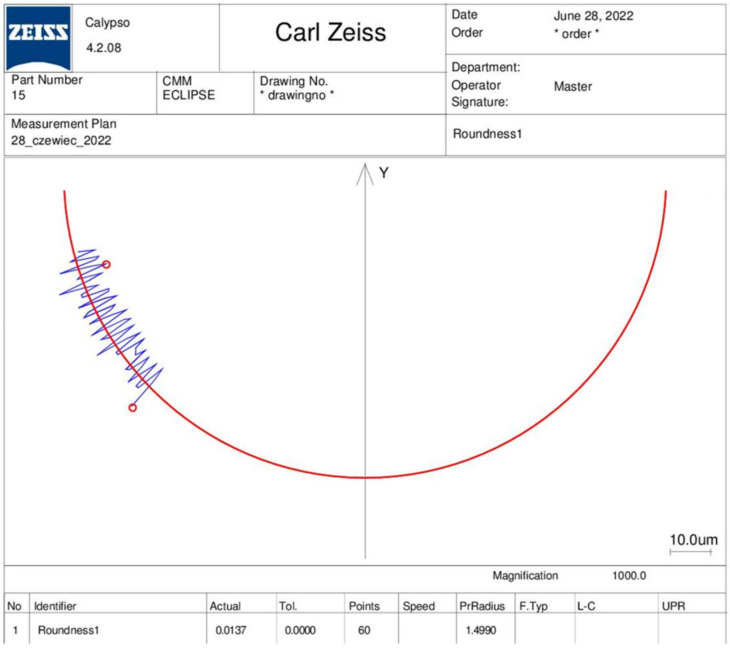
Measurement results of the deviation in the axial profile on CMM Zeiss Eclipse.

The average deviation value of the concave profile with the radius ρ=15 (mm) did not exceed 8 μm as the measurement value in the useful mating area of the helical surface. The maximum tolerance field of the measurement was 13 μm (see Figure 9). 

**Figure 10:**
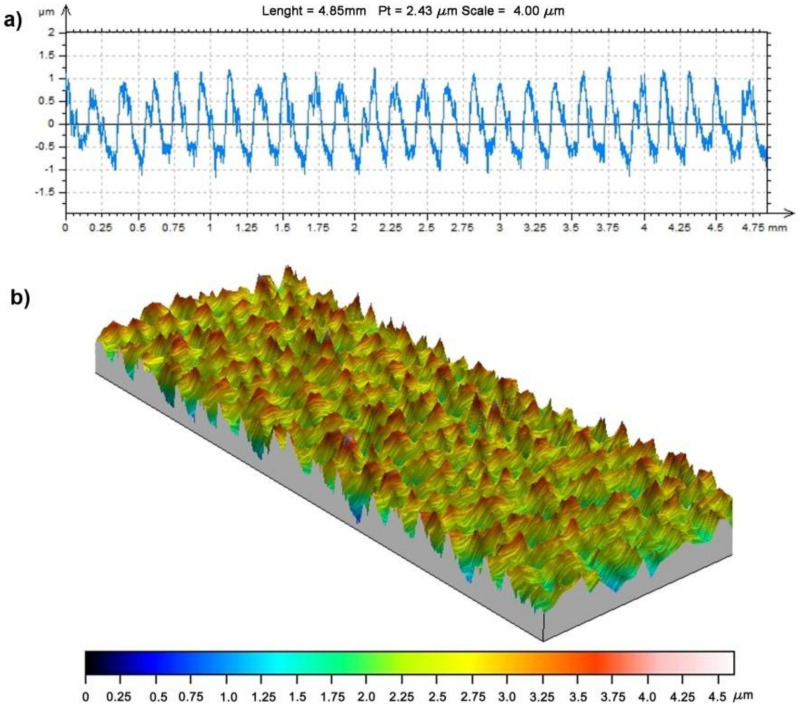
The thread flank after machining the worm with the parameters: *m* = 5, $d_{1}=48$ mm, and z1 = 1, using a ∅4 ball-end mill, and 50 milling passes: (**a**) surface-roughness profile; (**b**) spatial structure.

The accuracy of the worm concave profile was fully satisfactory, which resulted from correct theoretical assumptions, as well as the machining results. In addition to the thread profile measurement, a measurement of the roughness of the machined worm surfaces was taken. The measurement was conducted using a New Form Talysurf 120 2D/3D Taylor Hobson profilometer. Examples of the measurements of the lateral surface after machining a worm with a finger ball-end mill with a diameter of 4 mm developed in TalyMap Platinum (Taylor Hobson, New Star Road, UK, 2012) are shown in Figure 10.

The obtained roughness of the worm surface, as expressed by the Rz parameter, was between 2.0 and 3.23 µm. The roughness parameter (Rz) accurately reflects the local roughness changes, and hence, it was advisable to define it. The measurement was carried out perpendicular to the machining direction. Moreover, the roughness parameter (Ra) was measured in a similar way, and it was 1.0–1.48 μm. 

**Figure 11:**
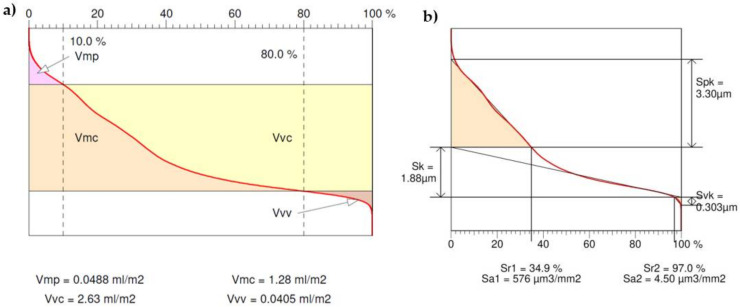
Abbott–Firestone curve (bearing area curve): (**a**) surface volumetric functional parameters; (**b**) position of the linearizing line of the curve (surface amplitude parameters).

Taking into account the functional parameters related to the volume, calculated on the basis of the material share (Figure 11a), the Abbott–Firestone curve, or bearing area curve (BAC), shows for a specific depth (vertical axis of the graph), the percentage of the material cut in relation to the coated material. The material share is expressed in multiples of the standard deviation, deposited evenly on the horizontal axis (see Figure 11a). From the left side, the curve is divided into five areas, depending on the zones of elevation formation [30]: inequalities: Zone 1—high peaks (or unexpected impurities); Zone 2—flattening area (stable); Zone 3—unstable transition area (transition from the flattened zone to the valley zone); Zone 4—dimple area; Zone 5—deep scratches and material defects. The following parameters were obtained: the peak volume of the surface material (Vmp) equal to 0.0488 mL/m^2^, the core volume of the surface material (Vmc) equal to 1.28 mL/m^2^, the volume of the hollow-core surface space (Vvc) equal to 2.63 mL/m^2^, and the cavity volume of the surface (Vvv) equal to 0.0405 mL/m^2^

## 4. Discussion

One of the most important requirements for the processes of shaping helical surfaces is the accuracy of the contour of the helical surfaces of the mating gear elements and the stereometric properties of the surfaces, which is because the load capacity and durability of the gear depend on them and the physical properties of the materials. Worms with a nominal circular-concave profile are characterized by very good conditions for the formation of oil films, which are conducive to obtaining a higher efficiency and higher load capacity. Worms of this type are difficult to produce with conventional technology because they require special tools for roughing and a properly profiled grinding wheel. This is because the outline of the tool is not an arc of a circle, and it also depends on the diameter of the grinding wheel and the position of its axis. Whenever the grinding wheel is sharpened, the outline changes because its diameter changes, and it requires difficult calculations and appropriate tooling for dressing the grinding wheel. 

The machining of steep surfaces, with a high inclination of the tool angle, which is a spherical cutter, is very often burdened with the high risk of the effect of the machining nature on the surface condition. It is well known that the most important factor for the roughness-amplitude parameters is the tool feed rate. In [31], the authors presented a model that additionally takes into account the analysis of the surface condition after machining such factors as the cutting displacement, chip-formation character, and acceleration of the cutting edge. Surface-texture analysis obviously requires a comprehensive approach in its nature towards the surface evaluation after machining. The analysis presented in this paper is a preliminary study, and it is an excellent introduction to a comprehensive assessment of the surface condition during the processing of worms with the ball-end mill cutter. Wojciechowski et al. [32] presented an analysis in this field, assessing the very success of the surface roughness for different tool inclination angles during the machining of the flat surfaces of hardened steels. When machining concave helical surfaces with the method of multiple passes, the tool is subjected to a strong phenomenon of vibration and deflection as a consequence, causing its axial runout. During the provided research, the phenomenon occurred, and in particular, in certain ranges of feeds and changes in the inclination of the material. In [33], the authors proposed a model of cutter displacement (vibration), including the tool geometry, cutting conditions, angle of inclination of the surface, and the runout and deflection of the tool (caused by cutting forces). The observations from the machining tests carried out confirm the authors’ results.

The multipassing method, which is commonly used for the machining of geometrically complex surfaces, can also be used for shaping worm helical surfaces with arbitrary axial profiles, with particular emphasis on concave shapes. Considering the large number of passes and the different tool positionings in each pass, the machining should be carried out on multiaxis CNC machine tools. In the case of shaping the helical surface of the worm with a spherical tool, the lateral surface of the worm consists of a plurality of helical partial surfaces that result from the multiple passes of the tool. Therefore, it seems necessary to use an appropriate number of tool passes in finishing machining. The side surface of a worm thread (thread profile) consists of many helical partial surfaces that result from multiple passes of the tool and are not closely related to the shape of the tool. This is a important advantage of this method, which gives the possibility of controlling the machining process in the entire finishing cycle. During the preparation of the machining, due attention was paid to the process of setting the tool, as well as to the accurate clamping of the blank part in the three-jaw chunk. The processing of three-parameter free surfaces is subject to very high requirements in this regard, and it is of particular importance in the processing of gears. In the analyzed case for a worm made with a circular-concave profile with a radius of 15 mm and a module of 5 mm, with the modification of the head radius and bottom transition curve with a rounding radius of 1 mm, the main surface was created by 50 passes of the tool on one side of the outline. The Vmp parameter determines the compliance of the surface during sliding and rolling lapping. In this case, the small value of this parameter indicates high abrasion resistance (i.e., good running-in behavior). In contrast, the void volume of a recess in the surface Vvv is a measure of the oil-holding capacity of the surface. 

The tribological properties of the surface can also be assessed by analyzing the bearing area curve (Figure 11b). The core roughness (Sk) is 1.88 µm, and it represents the roughness height after the initial lapping. The parameters Sr2 of the material share at the point where the core zone passes into the pit zone and Svk of the reduced valley depth allow for the assessment of the lubricating properties of the surfaces, and they are a measure of the ability to hold the fluid by the sliding surfaces. In this case, the Svk value is relatively small and amounts to 0.303 µm. 

## 5. Conclusions

The proposed machining method makes it possible to easily modify the outline of the worm, and, as a consequence, to modify the outline of the worm wheel. It allows the use of a universal tool, such as ball-end mill cutter, to make worms of any outline shape, and also with their modifications. This allows for the development of worm gears and the elimination of the limitations related to the technology of their manufacturing. The processing method is especially justified in the case of machining worms with large modules and nonstandard outline shapes (e.g., with circular profiles). It is an excellent alternative in the use of expensive and geometrically complex form tools. For the machined worm, a series of analyses was carried out, from which the following conclusions can be presented:

- Based on the measurements of the outline of the coordinate-measuring machine, it was found that, with the increase in the number of passes of the tool in the finishing machining, the conformity of the profile shape with the theoretical calculations increases, and it follows that the mean value of the deviation of the concave profile, with the radius ρ = 15 (mm), does not exceed 8 µm. The measurement tolerance field was a maximum of 13 μm. The distribution of the deviations in the circularity of the profile is uniform over the entire height of the profile of the useful worm coil;
- As a result of measuring the basic roughness parameters of the useful surface of a concave worm profile with a profilometer, the surface roughness determined by the Rz parameter was 2.0–3.23 µm. It should be noted that the measuring tip moved perpendicular to the machining direction. The measurement of the roughness parameter (Rz) was to assess the susceptibility to local changes in the roughness profile. The distribution of the above parameter was uniform over the entire surface area of the helical coil. Regarding the above remark, it should be noted that the machining was dry, and only a lubricant was added to the tool-acting area in small time intervals. The use of intensive cooling will certainly significantly improve the amplitude parameters of the quality of the surface-layer structure;
- For the Abbott–Firestone curve, the position of the linearizing line for the tested sample oscillates around 30 degrees, and such an inclination system of the material proportion curve proves the stability of the presence of the amount of material in the core of the surface layer. After milling with a finger milling cutter, the machined surface was characterized by a prism, and therefore, in this case, the values of the parameters Vvc and Vmc were quite large. In the case of high requirements and lowering these parameters, a slightly increased number of tool passes can be used. The value of the Spk parameter of the reduced peak height provides information on the wear resistance of the surface during cooperation. The size of the proportion (in comparison with the reduced valley depth (Svk)) of the parameter is large, and it indicates that the surface is susceptible to lapping, depending on the surface pressures up to a depth of 3 µm. Due to the very low Svk parameter, the core of the surface is stable, and it is not susceptible to changes due to surface pressures;
- The finishing of the concave surfaces that occurs on the side surfaces of the worm coils should be performed using in-cut milling. When using up-cut milling, surface tears occur at the place of the greatest arc of contact with the tool during machining;
- The method used is flexible in terms of the possibility of modifying the head or the fillet of the worm coils. The processing can then be carried out with the same tool. Technologically, this is a great simplification;
- The presented method of manufacturing can be treated as experimental. The presented results allow for the further development of worm machining with the use of CNC machine tools, with particular emphasis on machining nonstandard profiles.

## Data Availability

Data are contained within the article.
